# Associations between Screen-Based Sedentary Behaviour and Anxiety Symptoms in Mothers with Young Children

**DOI:** 10.1371/journal.pone.0155696

**Published:** 2016-05-18

**Authors:** Megan Teychenne, Trina Hinkley

**Affiliations:** Institute for Physical Activity and Nutrition (IPAN), School of Exercise and Nutrition Sciences, Deakin University, Geelong, Australia; Vanderbilt University, UNITED STATES

## Abstract

**Objectives:**

Anxiety is a serious illness and women (including mothers with young children) are at particular risk. Although physical activity (PA) may reduce anxiety risk, little research has investigated the link between sedentary behaviour and anxiety risk. The aim of this study was to examine the association between screen-based sedentary behaviour and anxiety symptoms, independent of PA, amongst mothers with young children.

**Methods:**

During 2013–2014, 528 mothers with children aged 2–5 years completed self-report measures of recreational screen-based sedentary behaviour (TV/DVD/video viewing, computer/e-games/hand held device use) and anxiety symptoms (using the Hospital Anxiety and Depression Scale, HADS-A). Linear regression analyses examined the cross-sectional association between screen-based sedentary behaviour and anxiety symptoms.

**Results:**

In models that adjusted for key demographic and behavioural covariates (including moderate- to vigorous-intensity PA, MVPA), computer/device use (B = 0.212; 95% CI = 0.048, 0.377) and total screen time (B = 0.109; 95% CI = 0.014, 0.205) were positively associated with heightened anxiety symptoms. TV viewing was not associated with anxiety symptoms in either model.

**Conclusions:**

Higher levels of recreational computer or handheld device use and overall screen time may be linked to higher risk of anxiety symptoms in mothers with young children, independent of MVPA. Further longitudinal and intervention research is required to determine temporal associations.

## Introduction

Mental health problems (which can include depression, anxiety and substance use disorders) are a leading cause of disability and mortality [1] and thus are a particular global public health concern. The global cost of mental health problems in 2010 was estimated to exceed US$ 2.5 trillion, with the cost predicted to rise to US$ 6.0 trillion by 2030 [2]. Current estimates suggest that 45% of adults in Australia [3] and 32% of adults in the US [4] will suffer from a mental health disorder in their lifetime, with anxiety disorders identified as the most common mental health issue amongst Australian [3] and US adults [4]. The cost of this illness impacts significantly on the public health system, families, as well as workplaces, with anxiety being linked to increased absenteeism and decreased productivity in the workplace [5]. Women are a high-risk group for developing anxiety, with women aged 25–34 years shown to be almost twice as likely to experience an anxiety disorder compared with men of the same age [3]. Further, the risk of anxiety has been shown to peak between the ages of 25–44 [3], which reflects the key childbearing years for women. Considering anxiety affects one’s quality of life and psychosocial functioning [6], with maternal anxiety also being shown to be a key predictor of child anxiety [7], it is important that strategies are identified to reduce the risk of anxiety in mothers.

Much evidence has suggested that physical activity (in particular that of which is undertaken during leisure-time) is beneficial for reducing anxiety symptoms [8,9]. However, mothers with young children (e.g. women with children aged under 5 years) often face great barriers to engaging in physical activity compared to the general population, and hence are more likely to be physically inactive [10]. Such barriers include lack of time and social support, and prioritising family commitments over their own personal well-being [11]. Thus, attempting to increase physical activity in mothers with young children may not be an effective means of reducing risk of anxiety. Additional behavioural strategies to reduce the risk of anxiety may need to be explored, such as reducing sedentary behaviour.

Sedentary behaviour is distinct from physical inactivity, being defined as sitting or reclining behaviours undertaken at or just above the resting metabolic rate [12]. Adults spend more than 50% of their waking time engaged in sedentary behaviour [13]. Although prevalence data for mothers specifically is limited, evidence shows that those aged 25–34 years (which is generally key childrearing age) spend the greatest amount of time in sedentary behaviour compared to any other age group. Television viewing and computer/internet use have been shown to be the most commonly reported sedentary behaviours amongst this age group [14]. Since parental screen time has been positively associated with preschool children’s screen time [15], targeting reductions in screen time amongst mothers may have positive impacts across two generations (i.e. mother and child).

A growing body of evidence shows that sedentary behaviour is linked to adverse health outcomes, including all-cause and cardiovascular disease mortality, independent of physical activity [16,17]. In terms of behaviour change, research has suggested that reducing sedentary behaviour may be viewed by adults as “easier” than increasing physical activity by adults, given that “lack of time” (the key barrier to being active) is not generally a barrier to reducing one’s sedentary behaviour [18], and that reducing sedentary behaviour is less physically demanding than increasing physical activity. Therefore, targeting reductions in sedentary behaviour may be more appealing and/or feasible for time-poor mothers with young children, compared to targeting increases in physical activity.

Sedentary behaviour has been recently positively linked to increased anxiety symptoms in the general population [19]. A systematic review of the association between sedentary behaviour and anxiety risk concluded that although there was a small amount of evidence to suggest a positive association between sitting time and anxiety risk, there was limited and inconsistent evidence for the relationship of other sedentary behaviours including screen time, television viewing time and computer use with anxiety risk [19]. For example, a previous study showed that television viewing and total screen-time were positively associated with anxiety symptoms, however, computer use was not [20]. In contrast, another study showed that computer use was positively associated with anxiety symptoms, however, TV viewing was not [21]. Given that individual sedentary behaviours (e.g. TV viewing, computer/device use) may have varying associations with anxiety symptoms due to the differences in contexts and purposes for engagement, it is essential to examine the link between individual sedentary behaviours, in particular screen-based entertainment such as television viewing and computer/device use, and anxiety risk.

No previous studies have investigated the link between more modern screen-based sedentary behaviours (i.e. tablet/device use) and anxiety risk, nor have any previous studies focussed on mothers with young children (a high-risk group for anxiety). Therefore, the aim of this study was to investigate the associations between screen-based sedentary behaviour (including TV/DVD viewing, and computer/e-games/device use) and anxiety symptoms, independent of physical activity, amongst mothers with young children.

Although research is currently limited and inconsistent regarding the link between screen-based sedentary behaviour (e.g. TV viewing, computer use) and anxiety risk, drawing on a larger and more consistent body of evidence indicating a positive association between screen-based sedentary behaviour and other mental health outcomes (particularly that of depression) [22], it was hypothesised that engagement in all screen-based sedentary behaviours would be associated with heightened anxiety symptoms, independent of physical activity, in the current study.

## Materials and Methods

This study utilised cross-sectional survey data collected in 2013 and 2014 from 575 mothers (with children aged 2–5 years) as a part of the “Mums, Dads and Kids Study”.

### Participants

Mothers of children aged 2–5 years were recruited via two pathways. Firstly, six local government areas (LGA’s) of varying levels of disadvantage (categorised as ‘low’, ‘medium’ and ‘high’ using the SEIFA index of advantage and disadvantage [23]) from within metropolitan Melbourne were randomly selected to be approached for recruitment. After approval was given to contact preschool and childcare centres managed by each of the LGA’s, approximately 15 facilities within each LGA were randomly selected to be contacted. Further providers that were not managed by the LGA but serving preschool-aged children (e.g. play groups, swimming schools, KinderGym) within the selected LGA’s were also approached. Relevant facilities/centres which served preschool-aged children from a seventh LGA (but who were not LGA-managed) were also invited to participate in the study in order to increase sample size. A total of 191 facilities were contacted and 140 (59 preschools/childcare centres and 81 other facilities) agreed to distribute information (via flyers, noticeboards, newsletters etc.) regarding the study to parents/guardians. Secondly, online blogs and *Facebook* pages related to parenting, families and child education were selected to be approached for recruitment, with 10 online blogs and 15 *Facebook* profile administrators agreeing to post information (e.g. flyers) online regarding the study.

Interested participants provided formal consent and were screened using an online survey to ensure they had at least one child aged two to five years who had not yet begun primary schooling. Participants that met these inclusion criteria were emailed a personalised link that directed them to the online survey site. A total of 1234 parents were screened and of those 911 (232 fathers, 679 mothers) were eligible and provided contact information to complete the survey. Of the mothers that were sent the survey link, a total of 24 participants data were not included in the study due to: 1) duplicate ID’s and/or partially complete data (n = 8); 2) the childs age was invalid or outside the age range (n = 13); 3) male sex (n = 1); and 4) impossible maternal age of <5 years and uncontactable to confirm correct date of birth (n = 2).

This left 655 respondents (mothers) with eligible data. Of those, 80 women were excluded because they reported being pregnant, were unsure of their pregnancy status, or did not complete this question. Thus the final sample consisted of a total of 528 women.

#### Ethics Statement

The study and consent procedure was approved by the Deakin University, Faculty of Health, Human Ethics Advisory Group (HEAG-H 138_2012) and the Department of Education, Employment and Childhood Development. Formal consent was obtained from all participants via checking an online box indicating their informed consent prior to completing the survey. Only participants that completed this step were able to then complete the survey.

### Measures

#### Independent variable

Screen-based sedentary behaviour was assessed using three measures. Firstly, participants were asked to estimate how much time they spend watching TV/DVD/videos on a typical weekday and weekend day. A weekly total was calculated by multiplying the duration of TV/DVD/Video watching on weekdays by five then adding this to the weekend days’ total duration (daily duration multiplied by two). A daily average was then calculated by dividing the weekly total by seven. Secondly, participants were asked to estimate how much time they spend using computers/electronic games/hand held devices for recreational purposes on a typical weekday and weekend day. Weekly totals and daily averages were calculated as above. Reliability of both measures was tested (Intra-class correlation [ICC] = 0.69 and 0.75 respectively). Thirdly, overall screen time was assessed by summing the reported daily average of time spent sitting watching TV/DVD/videos and time spent using computers/electronic games/hand held devices.

#### Dependent variable

Anxiety symptoms were measured using the well-validated anxiety sub-scale (HADS-A) of the Hospital Anxiety and Depression Scale (HADS) [24]. The anxiety sub-scale (HADS-A) includes seven items relating to symptoms of anxiety experienced in the past week. [25]. Each item has a 4-point Likert response scale, ranging from 0 (not at all) to 3 (most of the time/definitely). Anxiety symptoms were analysed as a continuous variable (with a possible range of 0–21). In terms of describing the sample, those that scored ≥8 on the scale were categorised as having heightened anxiety symptoms [24,25]. The HADS-A demonstrated good internal consistency (Cronbach’s = 0.82) in the current sample.

#### Covariates

Covariates were selected based on previous literature. Age, body mass index (BMI), education level, marital status, having a disability or poor health, and moderate- to vigorous-intensity physical activity (MVPA) were included in analyses as potentially confounding factors. MVPA was assessed using the Active Australia Survey [26] whereby participants reported the frequency and time (hours) they spend in a typical week undertaking both vigorous and moderate/walking physical activities in their free-time. Total weekly MVPA was calculated by multiplying the frequency and time spent in moderate-intensity physical activity and vigorous-intensity physical activity, and then summing these scores.

#### Missing data

The only variable with missing data was BMI (n = 5) and therefore these five cases were excluded from analyses in which BMI was an independent/dependent variable.

### Analyses

Descriptive univariate analyses were used to examine the distributions of socio-demographic characteristics, screen-based sedentary behaviours (hrs/day), MVPA (hrs/week) and anxiety symptoms. These analyses identified that all relevant variables were normally distributed. Crude and adjusted (controlling for sociodemographic covariates) linear regression models were used to test the associations between screen-based sedentary behaviours and anxiety symptoms. Linear regression analyses were also used to test for an interaction between overall screen-time, MVPA (categorised as either meeting the PA guidelines or not) and anxiety symptoms, in order to establish independence of association. MVPA was categorised for interaction analyses since dichotomising MVPA according to meeting guidelines is non arbitrary, provides guidance from a public health perspective and facilitates interpretation. Analyses were performed using STATA version 13.0.

## Results

Table 1 presents the socio-demographic characteristics among participants. The mean age of participants was 37.18 (SD = 4.62) years. Most women (95%) were married or in a de facto relationship, and nearly three quarters (74%) of the sample had completed a tertiary degree. Overall, 29.4% of women were experiencing anxiety symptoms.

**Table 1:** Socio-demographic characteristics of women (n = 528).

Characteristics	*N*	%
**Body mass index (BMI)**		
Healthy weight (<25)	307	58.70
Overweight (25–29.99)	144	27.53
Obese (≥30)	72	13.77
**Marital status**		
Married/defacto	500	94.70
Separated/divorced/widowed	28	5.30
**Education**		
Did not complete tertiary education	136	25.76
Completed tertiary education	392	74.24
**Having a disability or poor health**		
Yes	29	5.49
No	499	94.51
**Current work status (paid or voluntary)**		
<10 hours per week	223	42
10–30 hours per week	206	39
>30 hours per week	99	19
**Anxiety symptoms (HADS-A)**		
Not risk (HADS-A < 8)	373	70.64
At risk (HADS-A ≥ 8)	155	29.36
**Meeting PA recommendations**		
Met guidelines	391	74.05
Did not meet guidelines	137	25.95
	**Mean**	**SD**
Age	37.18	4.62
TV viewing (hours/day)	1.98	1.88
Computer/device use time (hours/day)	1.90	1.89
Overall screen time (hours/day)	3.88	3.27
MVPA (hours/week)	5.19	5.37

PA, Physical activity; MVPA, moderate-vigorous physical activity

Associations between screen-based sedentary behaviours and anxiety symptoms are presented in Table 2. In crude and adjusted models, computer/device use was positively associated with a higher likelihood of anxiety symptoms (B = 0.265 and 0.212 respectively). That is, in the adjusted model, every hour increase in computer/device use was associated with a 0.21 point increase in anxiety symptom score. A positive relationship was also shown for the association between overall screen time and anxiety symptoms in both crude and adjusted models (B = 0.131; B = 0.109 respectively). However, TV viewing was not associated with anxiety symptoms in either model.

**Table 2:** Linear regression analyses examining associations between sedentary behaviours and anxiety symptoms.

	Crude models			Adjusted models*		
Sedentary behaviour (hours/day)	B	95% CI	P value ^a^	B	Adjusted 95% CI	P value ^a^
TV viewing	0.127	-0.032, 0.288	0.117	0.109	-0.054, 0.274	0.188
Computer/device use	0.265	0.107, 0.423	**0.001**	0.212	0.048, 0.377	**0.011**
Overall screen time	0.131	0.040, 0.223	**0.005**	0.109	0.014, 0.205	**0.025**

^**a**^ Boldface indicates statistical significance (p<0.05)

*Models adjusted for age, education and body mass index (BMI), marital status, having a disability or poor health, moderate-vigorous physical activity (MVPA)

Interaction analyses showed no interactions between screen time, MVPA and anxiety symptoms in either the unadjusted or the adjusted models.

## Discussion

The current study builds on the very small existing body of research investigating the relationship between sedentary behaviour and anxiety symptoms. To date, no other studies have investigated the link between modern screen-based sedentary behaviours (i.e. tablet/device use) and anxiety symptoms, nor have studies focussed on mothers with young children. Our findings showed that certain screen-based sedentary behaviours (i.e. computer/hand held device use and overall screen time) were positively linked to anxiety symptoms in mothers with young children, independent of physical activity.

Specifically, this study found that higher levels of computer and/or hand held device use were associated with heightened anxiety symptoms in mothers with young children. This is consistent with findings from one other cross-sectional study that suggested that computer use was positively associated with anxiety symptoms in Australian adults [21]. One potential explanation for this relationship is that women often use computers/devices to utilise social media sites such as *Facebook*, *Instagram* and *Twitter*, which may have negative emotional and mental health effects. For example, one study has shown that nearly 50% of female *Facebook* users felt “addicted” to *Facebook*, 77% reported being online longer than they intend to be, and one quarter lost sleep because of *Facebook* [27]. This suggests that engagement in social media may lead to addictive tendencies and thus heightened symptoms of anxiety. However, since the current study did not measure social media usage specifically, the purpose of participants’ computer/device use is unknown and thus not necessarily spent on such sites, and hence other explanations are equally plausible. An alternate explanation for the positive relationship between computer/devise use and anxiety symptoms in mothers with young children is that the time spent using the computer/devise may remove them from other responsibilities such as household chores, engaging with their children, etc., which may subsequently lead to feelings of stress and anxiety. However, only two previous studies have investigated the association between computer use and anxiety risk [20,21], both of which did not assess modern screen-based sedentary behaviour (e.g. devise/tablet/smartphone use which was assessed in the current study) and thus a recent review of the literature concluded that there was insufficient data (i.e. only two previous studies with inconsistent findings) to show evidence of a relationship between computer use and anxiety risk [19]. Therefore, further research is needed to confirm the findings of the current study.

In the current study, overall screen time was also found to be linked to heightened anxiety symptoms. These findings are consistent with two previous cross-sectional studies which suggested that higher levels of screen time (i.e. TV and computer use collectively) were associated with increased anxiety symptoms in both adolescents [28] and adults [20]. In contrast to these findings, one previous study has shown an inverse association between screen time (i.e. combined TV, computer and electronic games use) and risk of anxiety, suggesting that greater engagement in screen-based entertainment was linked to lower levels of anxiety symptoms [29]. However, that study included a sample of 5-year old children and therefore the conflicting findings may be explained by the different target groups included in studies. It may be that screen time is more likely to impact on adult’s anxiety risk compared to children’s. Nevertheless, since only four observational studies (three cross-sectional and one longitudinal) have examined the link between screen time and anxiety symptoms [20,28,29,30], with inconsistent results (and none of which have focussed specifically on mothers), further longitudinal and intervention studies are needed to confirm findings from this study.

Parental anxiety has been linked to poor parenting practices (such as over maternal criticism [31]), lower quality child-parent interactions (e.g. anxious mothers are often less engaged and more withdrawn from their children [32]), poorer child behavioural and emotional outcomes (e.g. hyperactivity, conduct and emotional problems) [33] and a significantly increased risk of anxiety developing in their children [34]. Such outcomes (e.g. poor parenting practices, lower quality child-parent interactions, child behavioural/emotional problems) could also be hypothesised to be linked to parental screen-time, although the current body of literature is scarce. Thus, if findings of the current study are confirmed, implementing strategies such as reducing mother’s screen-based sedentary behaviour may have broad implications for families.

This study found no association between TV viewing and anxiety symptoms in mothers with young children. Only three previous cross-sectional studies have investigated the association between TV viewing and anxiety risk [20,21,35]. Our findings are consistent with one of those studies which was conducted in Australian adults [21]. Although two previous studies have found a positive association between TV viewing and anxiety symptoms [20,35], on balance there appears to still be insufficient evidence for the relationship between TV viewing and anxiety risk [19]. One such explanation for the lack of association between TV viewing and anxiety symptoms in the current study is that mothers may use TV viewing as a form of relaxation. This hypothesis is supported by one previous study amongst disadvantaged women with depressive symptoms, whereby TV viewing was suggested to be a tool women used to “switch off” and divert negative thoughts [36].

Finally, the current study showed that there was no interaction between screen time and physical activity, suggesting that sedentary behaviour was linked to risk of anxiety symptoms, independent of physical activity. Although no other studies have examined the potential interaction between sedentary behaviour, physical activity and anxiety risk specifically, one previous study has shown that physical activity was inversely linked to risk of a mental disorder (depression and anxiety combined), independent of sedentary behaviour [30]. Collectively, these results indicate that physical activity and sedentary behaviour may both be linked to risk of mental illness/anxiety symptoms; however, these relationships are likely to be independent of one another.

Limitations of this study should be acknowledged. Firstly, the study design was cross-sectional which does not allow for causality to be determined. For example, it is not known whether screen time predicts anxiety symptoms or whether anxiety symptoms predict screen time. Secondly, the sample was highly educated, with 75% of women having completed a University degree. Considering less educated adults are more likely to engage in leisure-time sedentary behaviours and be at risk of anxiety [37], further studies including a more representative sample of the population are needed. Nevertheless, analyses did control for education in this study to reduce the potential of such confounding relationships. Finally, only two leisure-time sedentary behaviours were assessed and therefore it is still unknown as to whether work or transport-related sedentary behaviours are linked to anxiety symptoms.

A key strength of this study is that it examined modern screen-based sedentary behaviours including device use (e.g. tablets and smartphones), which no other studies examining the association between sedentary behaviour and anxiety have done. Furthermore, this study controlled for a number of confounding factors including MVPA, which enabled the association between sedentary behaviour and anxiety symptoms, independent of physical activity, to be demonstrated.

### Conclusion

Considering economic costs of mental health problems, including anxiety, currently exceed US$ 2.5 trillion [2], and that anxiety significantly impacts on families as well as workplaces (e.g. increased absenteeism and decreased productivity amongst workers with anxiety symptoms) [5] it is vital that public health strategies are identified to reduce the burden of this increasingly prevalent mental health problem. In this study, higher levels of recreational computer or handheld device use and overall screen time was linked to heightened anxiety symptoms in mothers with young children, independent of MVPA. Although further longitudinal and interventional studies are required to confirm findings and to identify temporal associations, reducing the time that mothers with young children spend using computers and handheld devices for leisure purposes as well as overall screen time in leisure-time may be an important, yet cost-effective, strategy to lower the risk of anxiety in this high-risk target group.
